# The Impact of Job Insecurity and Distributive Injustice Post COVID-19 on Social Loafing Behavior among Hotel Workers: Mediating Role of Turnover Intention

**DOI:** 10.3390/ijerph19010411

**Published:** 2021-12-31

**Authors:** Mansour A. Alyahya, Ibrahim A. Elshaer, Abu Elnasr E. Sobaih

**Affiliations:** 1 Management Department, College of Business Administration, King Faisal University, Al-Ahsaa 31982, Saudi Arabia; malyahya@kfu.edu.sa (M.A.A.); asobaih@kfu.edu.sa (A.E.E.S.); 2 Faculty of Tourism and Hotels, Suez Canal University, Ismailia 41522, Egypt; 3 Faculty of Tourism and Hotel Management, Helwan University, Cairo 12612, Egypt

**Keywords:** social loafing, job insecurity, distributive injustice, turnover intention, COVID-19, Social Exchange Theory (SET), hotels

## Abstract

The COVID-19 pandemic has severe psychological and psychosocial impacts on hotel workers. This study examines the causal direct impact of both job insecurity and distributive injustice, which were common in hotels post COVID-19, on social loafing behavior among hotel workers, and the indirect impact through turnover intention. Data were collected from 850 hotels workers in the Kingdom of Saudi Arabia. Using results obtained through structural equation modeling (SEM), the spread of both job insecurity and distributive injustice positively and significantly influences turnover intention among hotel workers post the COVID-19 pandemic. The results also found that turnover intention fully mediates the influence of both distributive injustices on social loafing behavior. On the other side, it partially mediates job insecurity on social loafing behavior among hotel workers. Implications for scholars and practitioners as well as limitations of current research are discussed.

## 1. Introduction

The World Health Organization (WHO) reported the 2019 Novel Corona Virus Disease (known as COVID-19) as a worldwide pandemic in the first quarter of 2020. The virus, which was first reported from China in December 2019, has a fast spread and affected all countries rapidly. This worldwide pandemic heavily hit the international economy, and the tourism industry was among the hardest-hit industries [1]. Hence, many hotels were forced to lockdown, while others have had mass lay-offs [1]. The pandemic had severe psychological and psychosocial impacts on workers, especially those in the front lines [2]. However, there is limited published research examining the psychological influences of COVID-19 on hotel workers, despite that they are among the most affected workers due to the nature of the industry, which depends on people-to-people interaction. Previous research (e.g., [3,4]) has shown that crises, e.g., economic, political unrest, and health-related crises, often have a negative psychological impact on hotel workers and ultimately on their attitude and behaviors. Workers often feel stressed, unsecured, and worried about their continuity in the job and might think about changing their career [4,5,6,7,8]. The long lasting COVID-19 pandemic has helped the spread of the perception of job insecurity among hotels workers and has negatively affected their attitudes and behavior [8].

This research draws on Social Exchange Theory (SET) and examines the direct impact of job insecurity and distributive injustice post COVID-19 on social loafing behaviors among hotel workers and the indirect influence through turnover intention. The SET asserts that worker perceptions that s/he is supported or unsupported by his/her colleagues, supervisors, and organization result in reciprocal behavior towards his/her colleagues, supervisors, and organization [9]. Since the COVID-19 pandemic has had a severe psychological impact on workers [2], including hotel workers, this is worth investigation as well being undertaken by this research study.

Social loafing is defined as the tendency by an employee to put low efforts in groups or teamwork compared to efforts s/he spends individually [10,11]. Studies (see for example [12,13]) have confirmed several intrinsic and extrinsic factors that contribute to social loafing behavior. The extrinsic shape of social loafing occurs when an employee expects that his or her efforts are unidentifiable by others [13,14,15]. This means that if s/he feels that s/he will be unrewarded or receive sanction for efforts made, s/he is less likely to put efforts in groups [16]. On contrary, if s/he perceives his/her efforts are identifiable by their colleagues or supervisors, s/he is more likely to put efforts in groups and or teamwork [12]. The intrinsic shape of social loafing includes the beliefs by the employee that his/her efforts are meaningful and significant to the group and ultimately to organization overall [12].

Social loafing is a form of corrupt moral behavior, which not only impedes the work process by being inactive in the workplace but also reduces the motivation of other colleagues to engage in the group’s responsibilities [12]. The issue of social loafing cannot be underestimated in the work environment, because it can lead to severe consequences for the quality of productive work and progress of any organization [17]. Hence, it is crucial to understand and examine the causes and antecedence of social loafing behavior. This research examines the direct influence of distributive injustice and job insecurity on social loafing behavior and the indirect influence through turnover intention. Distributive injustice refers to employee perceptions that they are not given an equal share of organizational outcomes [18]. Job insecurity is the fear that employees have about continuity in their job and being unemployed [19]. This fear increased with the impact of COVID-19, which makes workers thinking about losing their jobs [5,8]. Turnover intention refers to employees’ thoughts about leaving the job [20].

Drawn on social theories, this research hypothesizes that both job insecurity and distributive injustice positively influence and increase social loafing behavior. Additionally, it is also expected that turnover intention post COVID-19 would have a mediating role in the impact of both distributive injustice and job insecurity on social loafing behavior. The research is among the new attempts that examine the psychological impact of COVID-19 on hotel workers and ultimately on their attitudes and behaviors. The research contributes to the academic body of hospitality literature and the practices of the hospitality industry in relation to the creation of an appropriate working environment in the new normal to achieve positive attitudes and behaviors among hotel employees.

## 2. Literature Review and Hypothesis Building

### 2.1. Job Insecurity and Turnover Intention

**Figure 1:**
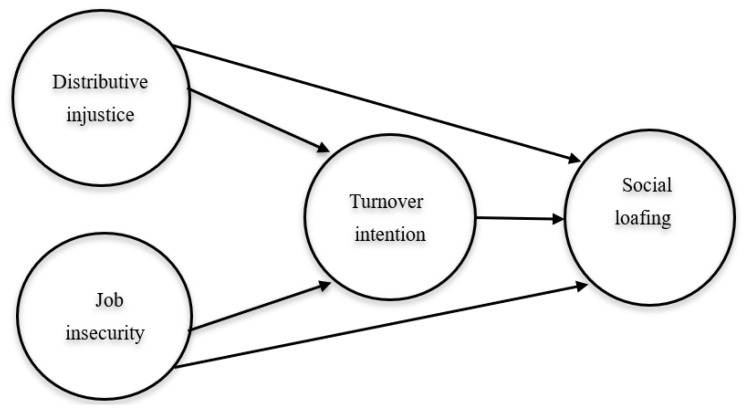
The research conceptual model.

Job insecurity is related to job stability and the concerns by workers that their job continuity is at risk [21]. This concern is defiantly induced by the surrounding work environment [22]. The uncertainty with pandemic has forced many hotels to mass lay-off, which has put millions of workers at risk [5,23]. Unsurprisingly, survivors, i.e., workers who stayed in the job during the crises, are concerned about their job continuity and are worried about their future, which has negative reactions such as the loss of morale and motivation, including undertaking unethical behaviors [8,24]. It is well-documented that employees who feel insecure in their jobs will decide to look for new jobs [5,25]. Research e.g., [26] has confirmed a positive association between job insecurity and job-seeking behaviors, confirming high turnover intention for those who feel high job insecurity. Additionally, recent research e.g., [5,8,27] showed that job insecurity is a predictor of turnover intention, which is the most common behavior with downsizing due to the impact of crises [4]. Based on these arguments and as seen in Figure 1, it could be hypothesized that:

**Hypothesis** **1** **(H1).** *Job insecurity positively influences turnover intention.*

### 2.2. Distributive Injustice and Turnover Intention

Distributive justice is one aspect of organizational justice and refers to the extent to which the organizational outcomes are distributed equally [28,29]. This includes wages, rewards, promotions social rights, and job outcomes. On the other side, distributive injustice occurs when an employee compares his/her job input to organizational outcomes against the performance of other employees with the feeling that there is inequity in the distribution of outcomes [30]. The inequity of this distribution is related to the poor relationship between supervisors and their employees [31].

Alam et al. [32] stated that if there was organizational justice in the distribution of tasks between workers and equality in the compensation system, the level of social loafing behavior between them would decrease. Mihelič and Culiberg [33] argued that the increase in the number of tasks without proper rewards increases the possibilities of social loafing and increases interdependence, and this leads to the dependence of some individuals on others and then this leads to the spread of this phenomenon among the group. The perceptions of injustice also make employees exhibit low job performance and decrease their collaboration with their colleagues [18]. Research showed that distributive injustice leads to employee unrest and causes job stress [34], which also may push them to think about leaving the job and searching for a new job. Based on these arguments, it could be hypothesized that:

**Hypothesis** **2** **(H2).** *Distributive injustice positively influences turnover intention.*

### 2.3. Turnover Intention and Social Loafing

Turnover intention is defined as the employee’s deliberate desire to quit his/her current job [35]. Hospitality is among the top industries that suffers from high turnover due to the low image of the industry with poor compensation [36,37]. Studies on employee turnover in the hospitality industry have shown several predictors for employee turnover intention, such as organizational support [38]; organizational citizenship behavior [39]; organizational justice [40]; job satisfaction and organizational commitment [41]; coworkers and job security [27]; and leadership style and organisztional commitment [42].

The social theory states that group members exhibit related behaviors because they monitor each other. Consequently, a group member who believes that other group members are not doing their best is likely to reduce his efforts equally [32]. If an individual’s effort was not specified in the group, the individual’s motivation would decrease, leading to social loafing [12,16]. According to Earley [43], social loafing is caused by low-performing workers who work as part of a group. Previous research e.g., [44] described social loafing as a decrease in individual energy due to the social presence of other individuals. Hoon and Tan [45] noted that social loafing is influenced by several important factors, including the individual’s personality traits, productive behavior, and general perceptions of continuing in their job. Recent research on restaurant employees confirmed a positive influence of turnover intention on social loafing behavior [27]. Based on this argument, it could be argued that:

**Hypothesis** **3** **(H3).** *Turnover intention positively influences social loafing.*

### 2.4. Job Insecurity and Social Loafing

As discussed earlier the SET explains workers’ behavior during social interaction, which takes a form of reciprocity [9]. This indicates that when a worker perceives positive practice or behavior from his colleague, supervisor, or organization, s/he returns by exhibiting positive behavior. Bultena [46] (1998) adopted SET to understand the influence of job insecurity on workers and their attitudes and behaviors. Thus, it is expected that workers’ perceptions of job insecurity, which they perceived post COVID-19 due to the influence of the severe pandemic on the hotel industry, may make them limit their efforts and their role behavior. Additionally, other studies e.g., [8] have confirmed that long lasting pandemic alerted job insecurity among hotel worker and hence they reported negative and unethical behavior as a result. Based on this argument, it could be argued that:

**Hypothesis** **4** **(H4).** *Job insecurity positively influences social loafing behavior.*

### 2.5. Distributive Injustice and Social Loafing

Based on Adams’ equity theory [29], if employees feel injustice, they respond with negative behavior towards their supervisors and other employees. West [47] commented on the percentage of fairness in the distribution of tasks in the group and argued that if the group produced equal results, the distribution of outcomes must be equal, and if the group considers the distribution to be unfair, this will affect their contribution to collective performance. Saad and Elshaer [31] argued that the compensation system should be consistent and equitable with workers’ behavior to promote a spirit of competition and enthusiasm. This promotes distributive justice, which includes perceived fairness regarding the compensations received [48]. Based on this discussion, it could be hypothesized that:

**Hypothesis** **5** **(H5).** *Distributive injustice positively influences social loafing behavior.*

### 2.6. The Mediating Effect of Turnover Intention

Jackson and Harkins [49] argued that social loafing behavior occurs because some colleagues do not feel the obligation to carry out their fair share of the tasks at work and have a sense of preference over other colleagues. This leads to administrative and organizational problems, bullying, and injustice. Additionally, the reward system has a major impact on social loafing behavior. Setting and applying a clear system for reward limit this phenomenon and motivates everyone for high performance [50]. Sparrowe et al. [51] studied relationship tasks and workers’ behaviors. The study indicated that the presence of social loafing decreases in simple tasks, but the presence of social loafing increases more in difficult and demanding tasks. Moreover, the expansion of the group numbers has a major impact on reducing performance in the end. Lee et al. [52] discussed the relationship between social loafing, the type of leadership, and negative psychological effects on the work environment. The results confirmed that supportive leadership has a positive impact on the organizational structure and general behavior.

Previous studies confirmed the direct influence of job insecurity on turnover intention [28] and the direct influence of distributive injustice on turnover intention [31]. Furthermore, the direct influence of turnover intention on social loafing [28]. This research assumes that both job insecurity and distributive injustice influence social loafing indirectly through turnover intention. This assumption is based on SET, which implies that if workers perceive injustice and feel insecure in their jobs, they respond by thinking about leaving the job and spending limited efforts collectively. This assumption is also supported by a previous research study on restaurant workers [28], which found that the mediating role between co-workers supports, job insecurity, and social loafing behavior is supported. Based on this discussion, it could be hypothesized that:

**Hypothesis** **6** **(H6).** *Turnover intention mediates the relationship between distributive injustice and social loafing.*

**Hypothesis** **7** **(H7).** *Turnover intention mediates the relationship between job insecurity and social loafing.*

## 3. Methodology

### 3.1. Measures

All the employed measures showed good psychometric properties and were derived from previous studies. The questionnaire used a multi-item scale (5 Likert scales) to measure the study constructs. Social loafing was measured by four items (a = 0.951) derived from Price, Harrison, and Gavin [53]. Respondents were asked to assess the level to which each fellow worker “loafed by not doing his or her share of the tasks, by leaving work for others to do, by loafing off, and by having other things to do when asked to help out”. Scores ranged on a 5-point scale, where 1 meant extremely likely to loaf and 5 reflected extremely unlikely to loaf. Colquitt’s [54] four-item scale of distributive justice was revised to measure distributive injustice (a = 0.954). A sample item covers “The outcome process does not reflect the effort I have put into my work”. The scale of job insecurity were derived from [55,56]. The scale has four items, including “I am not sure whether I shall achieve my career aims, I consider my professional development to be insecure”. The turnover intention was measured by three reflective items developed by [31,57,58] and reflects the intention to leave the career and find a new one. 

Nine academics and fifteen employees were asked to answer the questionnaire to pilot test it for clarity and reliability. No changes were made to the questionnaire content. The questionnaire declares clearly the anonymity and confidentiality of the collected data. Because the questionnaire was designed to be a self-reporting instrument, common method variance (CMV) may be an issue [59]. Harman’s single-factor analysis was conducted to deal with CMV, the extracted factors were constrained to the value of 1.00 in exploratory factor analysis (EFA) test using Statistical Package for the Social Sciences( SPSS) (IBM, NewYork, NY, USA)with no rotation approach. Only one factor emerged to explain 32 % (less than 50%) of the variance; accordingly, CMV is not a problem [60].

### 3.2. Data Collection

A randomly distributed questionnaire was directed to 550 full-time employees working in hotels in the Eastern Province in KSA (Emirate of the Eastern Province). It has the largest area among all the KSA Provinces and is exceptionally well-liked among visitors for its long beaches on the Persian Gulf. The questionnaire was distributed between January and February 2021. The research team uses its wide personal networks to drop and collect the relevant data, as this was the most effective method for obtaining a high response rate [61], in which 500 questionnaires were distributed, 455 returned, and 8 questionnaires were eliminated due to incomplete answers, yielding 447 valid questionnaires for analysis, with a response rate of around 89%. Our sample size of 447 is sufficient for SEM tests because it meets Nunnally’s [62] requirement of a minimum of ten respondents per item (our scale contains 15 indicators, so our sample size exceeds the required sample size of 150); it meets Boomsma’s [63] requirement of sample size based on the ratio of indicators (*p*) to latent variables (k), which in this study is 3.75 (15 indicators/four constructs) and thus requires a sampling size of 200 at least ; and, it meets Hair et al. [64] conditions of a minimum sample size between 100 to 150 to obtain good maximum likelihood estimation (MLE) solutions. Additionally, according to the Krejcie and Morgan [65] recommendations, if the population exceeds 1,000,000, the minimum required sample size is 384, in our study the sample size is 447, exceeding the recommendations. The independent sample *t*-test technique was used to examine the mean differences scores for early and late responses. Non-response bias was not a concern in this study, as there were no statistically significant differences between early and late responses (*p* > 0.05) [60].

## 4. Data Analysis

### 4.1. Descriptive Statistics

The majority of the respondents, 350 (57 percent) were male (78 percent) and married (83 percent). More than half (60 percent) were aged between 30 and 45 years. Around 51 percent were former college students. In terms of the service length, 325 respondents (73 percent) had worked for their company for less than five years, while 122 (27 percent) remained in service between 6 and 15 years.

Table 1 also includes some descriptive properties. The respondents’ mean (M) values ranged between 3.89 and 4.25, and the values of standard deviation (S.D) were between 0.769 and 1.081, revealing that the data were more spread and less focused around its mean value [66]. Table 2 also includes the skewness and kurtosis scores of data distribution, no values exceeded the score of −2 or +2, assuring the data normal distribution [67].

### 4.2. Confirmatory Factor Analysis (CFA)

All the independent and dependent dimensions with their related reflective items were subjected to first-order CFA with AMOS graphics to test the construct validity and reliability. The maximum likelihood (ML) estimation method was selected to evaluate the employed scale convergent and discriminant validity. As suggested by [67,68,69], various goodness of fit (GoF) criteria were employed to assess the fit of the model to data, including “normed chi-square” (chi-square divided by degree of freedom), “Comparative Fit Index” (CFI), “Tucker Lewis index” (TLI), “root means square error approximation” (RMSEA), and “standardised chi-square” (chi-square divided by degree of freedom) (PNFI). The Amos GoF results demonstrated that the CFA has a good model fit (see Table 3). The construct reliability was assessed using Cronbach’s alpha values (as previously mentioned) and composite reliability (CR). Table 3 shows that all the CR values exceeded the recommended cut-off point of 0.7 for all the four dimensions: social loafing (0.954), distributive injustice (0.956), job insecurity (0.951), and turnover intention (0.937), indicating that the data was internally consistent [67].

Moreover, the measuring scale convergent validity was successfully obtained for two main reasons: first, all of the reflective factor loadings (FL) were satisfactorily significant and high, as shown in Table 2. Table 2 shows that all FL values are between 0.805 and 0.971, exceeding the proposed cutoff value of 0.50 [67]. Second, all the average variance extracted (AVE) values, social loafing (0.838), turnover intention (0.832), distributive injustice (0.844), and job insecurity (0.830), exceeded the value of 0.50, showing satisfactory convergent validity [67] (see Table 2).

Additionally, the discriminant validity of the employed measures was obtained through following two main criteria as suggested [67,68,69]. The maximum shared variance (MSV) scores should be lower than the AVE scores, as shown in Table 2. Discriminant validity of the employed measures was obtained as well because the values of the square root of AVE for each individual dimension exceeded the values of dimensions intercorrelation with other dimensions (see Table 2).

### 4.3. Structural Equation Modeling (SEM)

**Figure 2:**
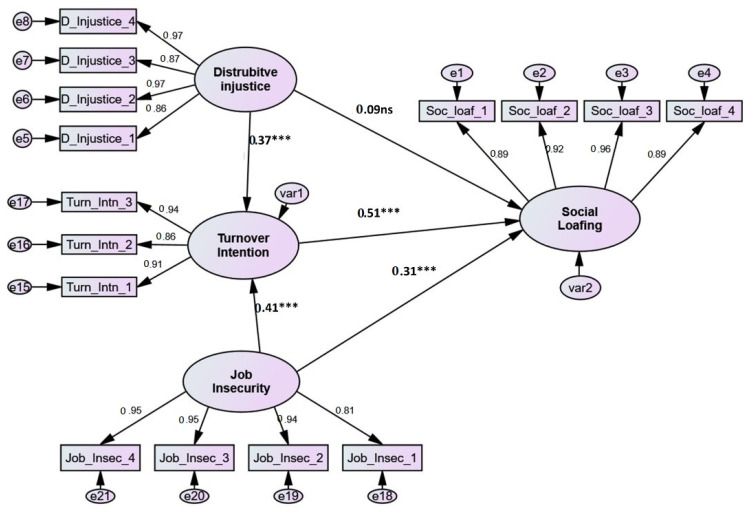
The structural mode. ***: *p* < 0.001.

This study took a confirmatory strategy, in which an extensive literature review was performed to establish a theoretical conceptual model, and then observed data was collected to see if it matched the previously specified theoretical conceptual model [68]. In this approach, the theoretical (structural) model is either rejected or authorised based on whether it meets a model fit condition. According to the SEM results, the structural model fit the data well: χ^2^ (85, N = 447) = 367.965, *p* < 0.001, (Normed χ^2^) =4.329, (SRMR = 0.044, RMSEA = 0.049, CFI = 0.953, NFI = 0.942, TLI = 0.941, PCFI = 0.771, and PNFI = 0.763). After obtaining adequate model fit to data, the study hypotheses were evaluated. The proposed hypotheses are pictured in Figure 2; each path in the picture is a specific hypothesis.

This research proposed seven hypotheses (five direct and two indirect). The first hypothesis that tests the impact of job insecurity on turnover intention (H1) is supported (*t*-value = 7.551, *p* < 0.001), with a highly significant standardized path coefficient of 0.41, revealing that job insecurity directly impacts turnover intention. Likewise, the SEM output shows that the effect of distributive injustice on turnover intention (H2) is significant and positive (*t*-value = 5.351, *p* < 0.001), with a path coefficient of 0.37; thus, hypothesis two (H2) is also supported. Similarly, the third hypothesis tested the impact of turnover intention on social loafing, and the SEM output proved a high positive and significant (*t*-value = 11.136, *p* < 0.001) impact, with a standardized path coeffective of 0.51, which support hypothesis three (H3). The impact of job insecurity on social loafing was found to be positive and significant (*t*-value = 4.295, *p* < 0.001) with a standardized path coeffective of 0.31, thus supporting hypothesis four (H4). However, the impact of distributive injustice on social loafing was found to be positive but insignificant (*t*-value = 1.944) with a standardised path coeffective of 0.09; thus, hypothesis five (H5) is not supported.

To examine the mediation effects, all paths were reviewed using recommendations from (1) Kelloway [70], for full and partial mediation requirements; (2) Zhao, Lynch, and Chen [71], for direct and indirect relationship conditions; and (3) Amos, for standardized direct/indirect effect. To identify the mediation, whether through standardizer regression or employing SEM, Zhao et al. [71] argued that only the indirect effects should be significant for the full mediation; however, if the direct and indirect relationships are both significant, partial mediations can be approved. Accordingly, the SEM output gives evidence that turnover intention completely mediates the relationship between distributive injustice and social loafing as the direct path is insignificant while the indirect two paths (from distributive injustice to turnover intention and from turnover intention to social loafing) are significant; thus, hypothesis six (H6) is supported. Similarly, turnover intention was found to partially mediate the relationship between job insecurity and social loafing, as the direct path and the indirect paths are significant, and thus hypothesis 7 (H7) is also supported [68]. The mediation effects (H6 and 7) can be further reinforced by exploring the direct and indirect results in AMOS output [29]. The direct positive insignificant impacts of distributive injustice on social loafing increased from (β = 0.09, *p* = 0.071) to a total effect of 0.25 with significant *p* > 0.001. Similarly, the direct positive significant impacts of job insecurity on social loafing increased from (β = 0.31, *p* > 0.001) to a total effect of 0.38 with *p* > 0.001. Table 3 shows as well that the explanatory power (R^2^) of all paths explains 31% of the variance in turnover intention (R^2^ = 0.41) and 34% of the variance in social loafing (R^2^ = 0.37).

The COVID-19 pandemic has had a severe impact on frontline workers, especially those in labor-intensive industries such as hotels. Drawn on Social Exchange Theory (SET), this research examined the direct impact of job insecurity and distributive injustice, which have become common practices amid COVID-19 in hotels on social loafing behaviors among hotel workers and the indirect influence through turnover intention. Seven research hypotheses were proposed. The first research hypothesis suggested a positive significant influence of job insecurity on turnover intention. As expected, the results supported this hypothesis and previous literature e.g., [4,7,8,27]. The COVID-19 pandemic has created a common perception of job insecurity among hotel workers, and this significantly affected their intention to leave the organization. Similarly, the results supported the second research hypothesis that distributive injustice positively and significantly influences employees’ turnover intention. This finding is supported by social theories and the norm of reciprocity between the organization from one side and employees on the other side that distributive injustice causes negative behavior, e.g., turnover intention [33].

The results confirmed a positive significant influence of both turnover intention and job insecurity on social loafing behavior which supported hypotheses three and four, respectively. Interestingly, the results showed that turnover intention was found to have the most significant stimulus of social loafing behavior among hotel workers. This reflects the value controlling all factors that lead to this intention in order to ensure positive consequences. On the other hand, the results failed to confirm the positive significant influence of distributive injustice on social loafing behavior. Hence, hypothesis five was not supported because the direct influence of distributive injustice on social loafing behavior is not significant. Hence, distributive injustice, perceived due to COVID-19 pandemic, is not a predicator of social loafing behavior. This reflects that other factors have a significant influence on social behavior amid COVID-19 than distributive injustice, e.g., job insecurity and turnover intention. This also means that despite hotel employees perceiving distributive injustice because of the COVID-19 pandemic, they did not respond directly by practicing social loafing behavior. However, they practice social loafing behaviors if they had an intention to leave the job.

The results supported both hypotheses six and seven in relation to the mediating effect of turnover in the relationship between distributive injustice and job insecurity and social loafing behavior individually. More specifically, turnover intention had a fully mediating role in the relationship between distributive injustice and social loafing behavior. This means that distributive injustice can only influence social loafing behavior through turnover intention, confirming that it is the only determining variable in this relationship. However, turnover intention had a partial mediating role in the relationship between job insecurity and social loafing behavior. This finding supports the previous literature review [27] that also found a mediating role in the relationship between job insecurity and social loafing behavior among restaurant workers, and also a recent study [E] that found a mediating role of turnover intention between job insecurity amid COVID-19 and unethical behavior among hotel workers.

These results have some implications for hospitality scholars and practitioners. The results of this research showed that social loafing behavior is an outcome of several antecedents, e.g., job insecurity and turnover intention in this research. Hence, for proper understanding and controlling this behavior, several stimuli of this behavior should be fully examined. Both scholars and practitioners should put more emphasis on the role of turnover intention, which becomes common post COVID-19, and promotes this negative behavior. Hospitality practitioners have to put more emphasis and investment on their workers to ensure their positive attitude and behavior, which impact the overall organizational performance. It is crucial that they recognize the value of their workers, especially during and after the crises. Hotel managers should make their best to ensure that their employees feel secure in their job and have no intention to leave the job to ensure positive outcomes. Manager should have direct communication with their employees and send them signals that they are the most important asset in the hotels, and they are valuable to the top management. Messages should be clearly disseminated to all employees that there is intention form management to lay-off workers, and that we are going to meet the pandemic together. Ensuring employee retention and eliminating turnover intention definitely decreases social loafing behavior. Additionally, hotels depend on teamwork culture, and hence, hotel practitioners should eliminate all stimuli of social loafing behavior to ensure proper teamwork in their workplace.

Another important implication of the current study for scholars is that despite the pandemic has spread the perceptions of job insecurity and distributive injustice, they can directly ensure negative behavior from employees. For example, the results of the current study should not significantly directly influence distributive injustice on social loafing behavior. This means that hotels employees do not care too much about distributive injustice amid COVID-19 in affecting their social loafing behavior and pay higher attention to other factors, e.g., job insecurity and turnover intention. The hardest-hit COVID-19 pandemic made employees prioritize staying in their current job and have less caring about distributive injustice amid the pandemic.

## 5. Limitations and Further Research

The study examined the direct impacts of both job insecurity and distributive injustice, which were common in hotels post COVID-19, on social loafing behavior among hotel workers and the indirect impact through turnover intention. The study was limited to full-time employees working in hotels in the Eastern Province in the Kingdom of Saudi Arabia, which may neither be representative of all hotels’ employees in KSA nor worldwide. Another research further opportunity could be to test the findings of the current research in a different context (countries and industries) with different types of employees, e.g., full-time and part-time, adopting multi-group analysis techniques to compare the results [72]. A cross-sectional sampling strategy was also used in the study. As a result, while potential causal relationships between the research factors might be deduced with care, they cannot be proven with certainty. Temporal ordering is one of the major requirements for confirmation [73]. Only a longitudinal study can establish temporal ordering; cross-sectional sampling alone will not suffice. In order to confirm the putative causal linkages in the current investigation, a long-term research design would be desirable.

## Figures and Tables

**Table 1:** Descriptive statistics.

Abbr.	Items	*N*	Min.	Max.	M	S.D	Skewness	Kurtosis
Social loafing (Price et al., 2006)								
Soc_loaf_1:	I left my work to others to do.	447	1	5	4.12	0.942	−1.365	1.048
Soc_loaf_2:	I claimed there were other things to do when others need help.	447	1	5	4.21	0.769	−0.939	1.190
Soc_loaf_3:	I avoided work and responsibility.	447	1	5	4.15	0.900	−1.320	1.274
Soc_loaf_4:	I loafed on my share of tasks.	447	1	5	4.10	0.917	−1.261	1.947
Distributive injustice (Colquitt 2001)								
D_Injustice1:	The outcome process does not reflect the effort I have put into my work.	447	1	5	3.89	0.972	−0.727	0.239
D_Injustice2:	The outcome process is inappropriate for the work I completed.	447	1	5	3.95	0.901	−0.842	0.464
D_Injustice3:	The outcome process does not reflect what I have contributed to the organization.	447	1	5	4.06	0.928	−0.842	0.220
D_Injustice4:	The outcome process is unjustified, given my performance.	447	1	5	3.95	0.920	−0.851	0.363
Turnover intention (Singh et al., 1996; Karatepe, 2009; Elshaer & Saad, 2017)								
Turn_Intn_1:	I often think about leaving that career.	447	1	5	4.00	1.075	−1.094	0.525
Turn_Intn_2:	It would not take much to make me leave this career.	447	1	5	3.99	1.079	−1.149	0.722
Turn_Intn_3:	I will probably be looking for another career soon.	447	1	5	3.99	1.081	−1.057	0.379
Job insecurity (Höge et al., 2012; Spurk et al., 2016)								
Job _Insec_1	I am not sure whether I shall achieve my career aims.	447	1	5	4.16	0.962	−1.561	1.655
Job _Insec_2	I consider my professional development to be insecure.	447	1	5	4.25	0.821	−1.239	1.537
Job _Insec_3	It is difficult for me to plan my professional future.	447	1	5	4.21	0.854	−1.338	1.295
Job _Insec_4	I often wonder how my career will develop.	447	1	5	4.24	0.844	−1.445	1.639

**Table 2:** Convergent and discriminant validity.

	Factors and Variables	Loading	CR	AVE	MSV	1	2	3	4
1-Social loafing (*a* = *0*.951)			0.954	0.838	0.076	**0.915**			
Soc_loaf_1	I left my work to others to do.	0.895							
Soc_loaf_2	I claimed there were other things to do when others needed help.	0.917							
Soc_loaf_3	I avoided work and responsibility.	0.959							
Soc_loaf_4	I loafed on my share of tasks.	0.888							
2-Distribtive injustice (*a* = *0*.954)			0.956	0.844	0.331	0.206	**0.918**		
Djustice1:	The outcome process does not reflect the effort I have put into my work.	0.856							
Djustice2:	The outcome process is inappropriate for the work I completed.	0.974							
Djustice3:	The outcome process does not reflect what I have contributed to the organization.	0.866							
Djustice4:	The outcome process is unjustified, given my performance.	0.971							
3-Turnover intention (*a* = *0*.936)			0.937	0.832	0.331	0.265	0.575	**0.912**	
Turn_Intn_1:	I often think about leaving that career.	0.916							
Turn_Intn_2:	It would not take much to make me leave this career.	0.871							
Turn_Intn_3:	I will probably be looking for another career soon.	0.948							
4-Job insecurity (*a* = *0*.947)			0.951	0.830	0.255	0.276	0.505	0.418	**0.911**
Job _Insec_1	I am not sure whether I shall achieve my career aims.	0.939							
Job _Insec_2	I consider my professional development to be insecure.	0.946							
Job _Insec_3	It is difficult for me to plan my professional future.	0.946							
Job _Insec_4	I often wonder how my career will develop.	0.805							

Model fit: (χ^2^ (84, N = 447) = 330.766, *p* < 0.001, normed χ^2^ = 3.938, RMSEA = 0.041, SRMR = 0.034, CFI = 0.968, TLI = 0.960, NFI = 0.958, PCFI = 0.775 and PNFI = 0.766). CR: composite reliability; AVE: average variance extracted; MSV: maximum shared value; diagonal values: the square root of AVE for each dimension; below diagonal values: intercorrelation between dimensions.

**Table 3:** Result of a structural model.

	Hypotheses					Beta (β)	C-R (*t*-Value)	R^2^	Hypotheses Results
H1	Job insecurity		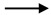	Turnover intention		0.41 ***	7.551		Supported
H2	Distributive injustice		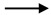	Turnover intention		0.37 ***	5.351		Supported
H3	Turnover intention		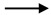	Social loafing		0.51 ***	11.136		Supported
H4	Job insecurity		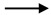	Social loafing		0.31 ***	4.295		Supported
H5	Distributive injustice		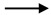	Social loafing		0.09	1.944		Not Supported
H6	Distributive injustice	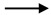	Turnover intention	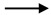	Social loafing	Path 1: β = 0.37 *** Path 2: β = 0.51 ***	Path 1: *t*-value = 5.351 Path 2: *t*-value = 11.136		Supported
H7	Job insecurity	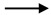	Turnover intention	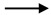	Social loafing	Path 1: β = 0.41 *** Path 2: β = 0.51 ***	Path 1: *t*-value = 7.551 Path 2: *t*-value = 11.136		Supported
Turnover intention								0.31	
Social loafing								0.37	

Model fit: (χ^2^ (85, N = 447) = 367.965, normed χ^2^ = 4.329, RMSEA = 0.049, SRMR = 0.044, CFI = 0.953, TLI = 0.941, NFI = 0.942, PCFI = 0.771 and PNFI = 0.763). ***: *p* < 0.001 5. Discussion and Implications.

## Data Availability

Data is available upon request from researchers who meet the eligibility criteria. Kindly contact the first author privately through e-mail.
